# Are we training psychiatrists to develop skills in intellectual disability psychiatry? Current European context and future directions

**DOI:** 10.1192/j.eurpsy.2020.102

**Published:** 2020-11-13

**Authors:** Marisa Casanova Dias, Bhathika Perera, Florian Riese, Livia De Picker, Mariana Pinto da Costa, Alina Petricean, Athanasios Kanellopoulos, Krzysztof Krysta, Franziska Baessler

**Affiliations:** 1 National Centre for Mental Health, School of Medicine, Cardiff University, Cardiff, United Kingdom; 2 Section of Women’s Mental Health, Institute of Psychiatry, Psychology and Neurosciences, King’s College London, London, United Kingdom; 3 Haringey Learning Disability Partnership, Barnet, Enfield and Haringey Mental Health Trust, London, United Kingdom; 4 Psychiatric University Hospital Zurich, Zurich, Switzerland; 5 Collaborative Antwerp Psychiatric Research Institute, University of Antwerp, Antwerp, Belgium; 6 University Psychiatric Hospital Campus Duffel, Antwerp, Belgium; 7 Unit for Social and Community Psychiatry, WHO Collaborating Centre for Mental Health Services Development, Queen Mary University of London, London, United Kingdom; 8 Institute of Biomedical Sciences Abel Salazar, University of Porto, Porto, Portugal; 9 Hospital de Magalhães Lemos, Porto, Portugal; 10 National Centre for Mental Health, BSMHFT, Birmingham, United Kingdom; 11 Center for Adolescent Medicine, First Department of Pediatrics, School of Medicine, National and Kapodistrian University of Athens, Aghia Sophia Children’s Hospital, Athens, Greece; 12 Department of Rehabilitation Psychiatry, Medical University of Silesia, Katowice, Poland; 13 Department of General Internal and Psychosomatic Medicine, Heidelberg University Hospital, Heidelberg, Germany

**Keywords:** Community Mental Health Teams, education and training, intellectual disability, learning disability, specialty training

## Abstract

The majority of people with intellectual disabilities (ID) and psychiatric disorders access mainstream mental health services across Europe. However, only 56% of countries provide postgraduate psychiatric training in ID according to a survey across 42 European countries. We explore the challenges of ID training and make recommendations for education and health policymakers.

Intellectual disability (ID) is a neurodevelopmental disorder affecting 1–2% of the population [1]. Current estimates suggest that there are nearly 7 million people with ID in Europe.

It is well established that people with intellectual disabilities have significantly higher rates of mental [2] and physical health disorders [3] as well as social care needs which must be considered alongside one another. This means that they require high and complex multidisciplinary service provision with an estimated cost of over € 43 billion each year in Europe.

However, the provision of services is often poorly planned and does not necessarily match the needs of people with ID [4–6]. This is not only due to economic constraints, but also due to a more systemic problem of unavailability of community services and skilled professionals. The WHO Atlas on global resources for persons with ID [4] reports that the percentage of countries in Europe reporting specific training in ID for psychiatrists was 46.8%. The other most trained health professionals were: special educators (59.6%), speech and language therapists (44.7%), psychologists (38.3%), and pediatricians (21.3%). It is not surprising that the majority of countries train other health professionals, as much of the work requires a multidisciplinary team. A review of ID training in health sciences across Europe found that studies in this field are scarce in Europe compared with other parts of the world [7]. The only survey done so far that covered postgraduate psychiatry ID training was done in 2004 and only covered 22 countries, out of which only 14 (64%) reported structured ID training [8].

Without qualified and skilled clinicians to look after people with ID, the care and support of people with ID cannot be further improved. Given mental health problems are highly prevalent in people with ID [2], there is a case to re-evaluate current mental health care provisions and skilled staff to manage this group of individuals with multiple vulnerabilities.

## The Importance of Postgraduate Psychiatric Education on ID

Qualified clinicians are an essential pillar to improved care. The content and methods of postgraduate psychiatric training profoundly shape future psychiatrists and the quality of psychiatric care and expertise [9].

In Europe, the international frameworks for training standards, including ID, are issued by the Union Européenne des Médecins Spécialistes (UEMS). Despite these harmonized evidence-based policies, for example “Training Requirements for the specialty of Psychiatry” (uemspsychiatry.org), and the automatic mutual recognition of qualification in EU, the practice differs vastly [10].

Each person, regardless of the country they live in, has the right to health, to have the best care possible, and to have it delivered by skilled professionals.

## A European Survey of Postgraduate Psychiatry Training Across 42 Countries

The European Federation of Psychiatric Trainees (EFPT, www.efpt.eu), an umbrella organization for trainee associations, conducted the most comprehensive study so far of postgraduate psychiatric training and provision of ID training across Europe.

In this cross-sectional study, the online questionnaire was sent to the representatives of national psychiatric trainee associations, or trainees with comparable functions and with access to accurate and comprehensive data on national practices in countries without such associations. Topics covered included the general structure and length of psychiatric postgraduate training and specific questions about the availability and provision of ID training.

Out of the 42 countries surveyed in 2014, 41 (98% response rate) answered the questions on the availability of ID training (see Table 1). Twenty-three (56%) of the countries provided postgraduate psychiatric training on ID and 18 (44%) did not, of which, one did not have a training scheme of their own. Where training was provided, it took several formats such as lectures, clinical rotations, and case discussions (Table 1). In many countries, ID training was linked to child and adolescent psychiatry training. In two countries, Ireland and the United Kingdom, ID is recognized as a subspecialty with ID training lasting 3 years. This subspecialty training takes place after 3 years of core training in psychiatry (total specialty training of 6 years). In other countries, the length of ID training varied from 6 months, offered as a clinical rotation, to merely hours in those countries offering lectures only. Psychiatry training programs varied considerably in length, from 7 years in Ireland to 1 year in Belarus. In Belarus, despite having the shortest training program, a mandatory 2-week clinical rotation in “behavioral and emotional disorders with onset usually occurring in childhood and adolescence” is provided.

**Table 1.** Intellectual Disability Psychiatry training offered across 42 European countries

Country	Intellectual disability psychiatry as part of psychiatric training	Teaching format	Nationally standardized training program	Duration of adult psychiatry training (years)	Duration of child and adolescent psychiatry training (CAP) (years)
Albania	No	–	NR	4	NR
Austria	No	–	Yes	6	6
Azerbaijan	Yes	Lectures	Yes	2	n.a.
Belarus	Yes	Lectures, clinical rotation (2 weeks), case	Yes	1	1
Belgium	Yes	Mandatory lectures, optional clinical rotation (6 months)	No	5	5
Bosnia Herzegovina	NR	NR	No	5	NR
Bulgaria	Yes	Part of CAP clinical rotation (4 months)	Yes	4	4
Croatia	No	–	Yes	5	5
Cyprus	No	–	NR	5	NR
Czech Republic	Yes	–	NR	5	NR
Denmark	Yes	Very few lectures, probably less than 2 h	Yes	5	5
Estonia	Yes	Lectures, CAP training (6 months)	Yes	4	5
Finland	Yes	In CAP, it is possible to have clinical rotation in child neurology (6 months)	No	6	6
France	No	–	No	4	4
Georgia	No	–	Yes	4	n.a.
Germany	Yes	Lectures and optional rotation	Yes	5	5
Greece	Yes	Lectures	No	5	5
Hungary	No	–	Yes	5	5
Ireland	Yes	Learning outcomes for psychiatry of intellectual disability must be attained. In practice it will not be possible to achieve outcomes in all specialties with a clinical attachment; therefore, doing an attachment in intellectual disability is not mandatory for BST. Where this is not provided by clinical attachment the learning outcomes must be addressed through other methods (e.g., a combination of courses, workshops, seminars, specialist clinic attendance, e-learning, etc.). 3 years clinical training in HST is required for certification in intellectual disability (or 2 years in dual certification)	Yes	7	7
Italy	No	–	Yes	5	5
Israel	Yes	Lectures, case reviews	Yes	4	5
Latvia	Yes	NR	Yes	5	NR
Lithuania	No	–	Yes	4	4
Luxembourg	n.a.	–	–	–	–
North Macedonia	Yes	Lectures	Yes	5	–
Malta	Yes	Lectures and clinical rotation (3 months)	Yes	4	1
Montenegro	Yes	Lectures		4	
Norway	No	–	Yes	5	5
Poland	No	–	Yes	5	5
Portugal	No	–	Yes	5	5
Romania	No	–	Yes	4	4
Russia	Yes	Lectures, examinations (mandatory), rotations (optional)	Yes	2	n.a.
Serbia	Yes	Lectures and clinical practice	Yes	4	4
Slovakia	No	–	Yes	5	5
Slovenia	No	–	Yes	5	5
Spain	Yes	–	NR	4	n.a.
Sweden	Yes	Lectures and clinical rotation	Yes	5	5
Switzerland	No	–	Yes	6	6
The Netherlands	No	–	Yes	5	3
Turkey	Yes	Lectures, outpatient practice, forensic psychiatry	Yes	4	4
Ukraine	Yes	Lectures	Yes	1	2
United Kingdom	Yes	This is taught as part of the compulsory MRCPsych exam preparation course and is tested in the exam. Additionally, some trainees may complete an ID placement (6 months) during core training. There is also the possibility of completing the 3-year higher specialty training program in ID.	Yes	6	6

This information is correct at the time of collection (2014–2015). The authors are not aware of significant changes to psychiatric training programs since then. The information provided reflects the actual experiences of trainees about the provision of training. In countries where training is not nationally standardized, there may be bigger variation within different regions of the same country. There is no psychiatric training program in Luxembourg.

Abbreviations: n.a., not applicable; NR, no response; BST, Basic Specialist Training; HST, Higher Specialist Training; MRCPsych, Membership of the Royal College of Psychiatrists.

The design of this study, where the respondents have the responsibility of being national representatives, ensured an extremely high response rate (98%) and assures us of the accuracy of the data. Furthermore, the information provided reflects the actual experiences of trainees about the provision of training. In countries where training is not nationally standardized there may be bigger variation within different regions of the same country that could not be captured, although these respondents would be the best placed to have such intelligence. The authors are not aware of significant changes to psychiatric training programs since the time of data collection.

To our knowledge, this is the most recent and comprehensive study on this topic. The only other survey done so far that covered postgraduate psychiatry ID training was done in 2004 and was much smaller [8]. It showed that 14 out of the 22 (64%) countries surveyed offered structured theoretical training in their national training scheme and 5 (23%) offered mandatory practical training, although it did not specify which countries, so we cannot draw a comparison. That study recommended intellectual disabilities, leadership and management, informatics and telemedicine should be introduced in the training curricula, but we have not seen much progress in over 10 years.

## The Outlook of ID Psychiatry in Europe

### Challenges

The biggest question is whether we need specialists in ID psychiatry.

In many countries, less time available for training and constraints of service provision during training limit the exposure to ID. Limited exposure to people with ID and their problems contributes to increase the stigma these people already face. Previous exposure, either in personal or working life, has shown to influence choice of ID subspecialty training. Different modalities of training operate in Europe with a debate about the need for a subspecialty in ID versus the need for all psychiatrists to have ID training. The former has been criticized alongside other subspecialties as it can lead to fragmentation of care. The latter, despite great efforts, has yet to be implemented within and between countries.

The cost of ignorance on the subject matter due to lack of training can be far-reaching. Firstly, diagnosing mental disorders in people with ID can be challenging for many reasons. There is the risk of under-diagnosing or mis-diagnosing mental disorders in people with ID. The presence of multiple physical, neurodevelopmental, and psychiatric co-morbidities can change the way mental illnesses present in people with ID. Diagnostic overshadowing is very commonly reported. Lower intellectual functioning along with communication difficulties can affect how psychiatric symptoms are manifested. Failing to identify and treat early can have an adverse outcome in health leading to premature death and social disintegration. Secondly, lack of skills to identify the real issues and lack of knowledge or availability of specialist multidisciplinary centers lead to unwarranted and off-label prescription of medication, for example, antipsychotics with the deleterious consequences associated with those, or to over-use of physical restraint. Thirdly, the lack of skilled adult psychiatrists leads on many occasions to child and adolescent psychiatrists being approached by parents of people with ID already in their 20s because they are desperate and have no-one to turn to with sufficient expertise.

### Recommendations for education and health policymakers

• Given the above, the authors believe ID psychiatry should be a mandatory part of training curricula and exams (assessment drives learning) in all European countries.
• We acknowledge the different challenges faced by different countries, related to economic power, organization of services, organization of training, provision of social care, and general societal expectations. Nonetheless, we suggest that through harmonized training—between and within countries—we can promote harmonized care despite all the existing contextual differences [10].
• Professional exchange programs, like those provided by EFPT and EPA offer a means to complement the training curricula where opportunities are not available locally.
• Importantly, even after the completion of postgraduate training, the provision of quality Continuous Medical Education (CME) on the topic, especially through new technologies, can increase the reach of ID training, overcoming location and economic barriers.
• We recommend that ID training should not be limited to CAP training, but should work on facilitating the transition between CAP, when the disorders are normally identified, adult psychiatry, as well as old age psychiatry—as the life-expectancy of people with ID continues to increase.
• Last but not least, it is important to remember that improvements in the field will come from good cooperation with other professionals and including people with ID, their families, and carers as stakeholders.

National and international policy is an important lever for directing and effecting change. Without improvements in postgraduate psychiatric training in ID, psychiatric care for people with ID will not fundamentally improve. Looking at the survey results, not much has happened in the past decade. For the benefit of people with ID, we need to come together to make a more active push for curriculum reform across Europe.

## Data Availability

The data that support the findings of this study are available from the corresponding author, M.C.D., upon reasonable request.

## Supplementary material

For supplementary material accompanying this paper visit https://doi.org/10.1192/j.eurpsy.2020.102. click here to view supplementary material
