# Supplementary material for: Are we training psychiatrists to develop skills in intellectual disability psychiatry? Current European context and future directions
Source: Eur Psychiatry. 2020 Nov 13;63(1):e99. doi: 10.1192/j.eurpsy.2020.102 (PMC7737176; doi:10.1192/j.eurpsy.2020.102)
Supplement: Supplementary file 1 [file S0924933820001029sup001.docx]

Figure 1. Intellectual Disability Psychiatry training offered across 42 European countries
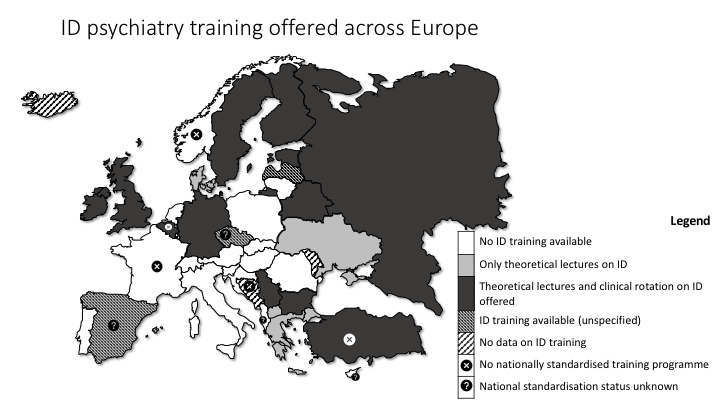


| **Country** | **Intellectual Disability Psychiatry as part of psychiatric training** | **Teaching format** | **Nationally standardised training programme** | **Duration of Adult Psychiatry training**  **(years)** | **Duration of Child and adolescent Psychiatry training (CAP)**  **(years)** |
| --- | --- | --- | --- | --- | --- |
| Albania | No | - | NR | 4 | NR |
| Austria | No | - | Yes | 6 | 6 |
| Azerbaijan | Yes | Lectures | Yes | 2 | n.a. |
| Belarus | Yes | Lectures, clinical rotation (2 weeks), case | Yes | 1 | 1 |
| Belgium | Yes | Mandatory lectures, optional clinical rotation (6 months) | No | 5 | 5 |
| Bosnia Herzegovina | NR | NR | No | 5 | NR |
| Bulgaria | Yes | Part of CAP clinical rotation (4 months) | Yes | 4 | 4 |
| Croatia | No | - | Yes | 5 | 5 |
| Cyprus | No | - | NR | 5 | NR |
| Czech Republic | Yes | - | NR | 5 | NR |
| Denmark | Yes | Very few lectures, probably less than two hours | Yes | 5 | 5 |
| Estonia | Yes | Lectures, CAP training (6 months) | Yes | 4 | 5 |
| Finland | Yes | In CAP, it is possible to have clinical rotation in child neurology (6 months) | No | 6 | 6 |
| France | No |  | No | 4 | 4 |
| Georgia | No |  | Yes | 4 | n.a. |
| Germany | Yes | Lectures and optional rotation | Yes | 5 | 5 |
| Greece | Yes | Lectures | No | 5 | 5 |
| Hungary | No |  | Yes | 5 | 5 |
| Ireland | Yes | Learning outcomes for Psychiatry of Intellectual Disability must be attained. In practice it will not be possible to achieve outcomes in all specialties with a clinical attachment; therefore, doing an attachment in intellectual disability is not mandatory for BST. Where this is not provided by clinical attachment the learning outcomes must be addressed through other methods (e.g. a combination of courses, workshops, seminars, specialist clinic attendance, e-learning, etc.). 3 years Clinical training in HST is required for certification in intellectual disability (or 2 years in dual certification) | Yes | 7 | 7 |
| Italy | No |  | Yes | 5 | 5 |
| Israel | Yes | Lectures, case reviews | Yes | 4 | 5 |
| Latvia | Yes | NR | Yes | 5 | NR |
| Lithuania | No | - | Yes | 4 | 4 |
| Luxembourg | n.a. | - | - | - | - |
| North Macedonia | Yes | Lectures | Yes | 5 | - |
| Malta | Yes | Lectures and clinical rotation (3 months) | Yes | 4 | 1 |
| Montenegro | Yes | Lectures |  | 4 |  |
| Norway | No | - | Yes | 5 | 5 |
| Poland | No | - | Yes | 5 | 5 |
| Portugal | No | - | Yes | 5 | 5 |
| Romania | No | - | Yes | 4 | 4 |
| Russia | Yes | Lectures, examinations (mandatory), rotations (optional) | Yes | 2 | n.a. |
| Serbia | Yes | Lectures and clinical practice | Yes | 4 | 4 |
| Slovakia | No |  | Yes | 5 | 5 |
| Slovenia | No |  | Yes | 5 | 5 |
| Spain | Yes |  | NR | 4 | n.a. |
| Sweden | Yes | Lectures and clinical rotation | Yes | 5 | 5 |
| Switzerland | No |  | Yes | 6 | 6 |
| The Netherlands | No |  | Yes | 5 | 3 |
| Turkey | Yes | Lectures, outpatient practice, forensic psychiatry | Yes | 4 | 4 |
| Ukraine | Yes | Lectures | Yes | 1 | 2 |
| United Kingdom | Yes | This is taught as part of the compulsory MRCPsych exam preparation course and is tested in the exam. Additionally, some trainees may complete an ID placement (6 months) during core training. There is also the possibility of completing the three-year higher specialty training programme in ID. | Yes | 6 | 6 |

n.a. *not applicable*, NR *No Response*; This information is correct at the time of collection (2014-2015). The authors are not aware of significant changes to psychiatric training programmes since then. The information provided reflects the actual experiences of trainees about the provision of training. In countries where training is not nationally standardised there may be bigger variation within different regions of the same country. There is no psychiatric training programme in Luxembourg.
